# Have inequalities in all-cause and cause-specific child mortality between countries declined across the world?

**DOI:** 10.1186/s12939-019-1102-3

**Published:** 2019-12-31

**Authors:** Seungman Cha, Yan Jin

**Affiliations:** 1 Department of Global Development and Entrepreneurship, Graduate School of Global Development and Entrepreneurship, Handong Global University, Pohang, 37554 South Korea; 2 Department of Disease Control, Faculty of Infectious and Tropical Disease, London School of Hygiene & Tropical Medicine, Keppel Street, London, WC1E 7HT UK; 3 Department of Microbiology, Dongguk University College of Medicine, Dongdaero 123, Gyeongju, Republic of Korea 38066

**Keywords:** Child mortality, Inequality, Gross domestic product per capita, Neonatal mortality, Post-neonatal mortality, Cause-specific child mortality, Time trend

## Abstract

**Background:**

Comparing the distribution of all cause or cause-specific child mortality in countries by income and its progress over time has not been rigorously monitored, and hence remains unknown. We therefore aimed to analyze child mortality disparities between countries with respect to income level and progression for the period 2000–2015, and further explored the convergence of unequal income levels across the globe.

**Methods:**

Four types of measures were used to assess the degree of inequality across countries: difference and ratio of child mortality rate, the concentration index, and the Erreygers index. To assess the longitudinal trend of unequal child mortality rate by wealth ranking, hierarchical mixed effect analysis was used to examine any significant changes in the slope of under-5 child mortality rate by GDP per capita between 2000 and 2015.

**Results:**

All four measures reveal significant inequalities across the countries by income level. Compared with children in the least deprived socioeconomic quintile, the mortality rate for children in the most deprived socioeconomic quintile was nearly 20.7 times higher (95% Confidence Interval: 20.5–20.8) in 2000, and 12.2 times (95% CI: 12.1–12.3) higher in 2015. Globally, the relative and absolute inequality of child mortality between the first and fifth quintiles have declined over time in all diseases, but was more pronounced for infectious diseases (pneumonia, diarrhea, measles, and meningitis). In 2000, post-neonatal children in the first quintile had 105.3 times (95% CI: 100.8–110.0) and 216.3 times (95% CI: 202.5–231.2) higher risks of pneumonia- and diarrhea-specific child mortality than children in the fifth quintile. In 2015, the corresponding rate ratios had decreased to 59.3 (95% CI: 56.5–62.1) and 101.9 (95% CI: 94.3–110.0) times. However, compared with non-communicable disease, infectious diseases still show a far more severe disparity between income quintile. Mixed effect analysis demonstrates the convergence of under-5 mortality in 194 countries across income levels.

**Conclusion:**

Grand convergence in child mortality, particularly in post neonatal children, suggests that the global community has witnessed success to some extent in controlling infectious diseases. To our knowledge, this study is the first to assess worldwide inequalities in cause-specific child mortality and its time trend by wealth.

## Background

Narrowing the gaps in child mortality is the core concern of the global health community [1, 2]. Upholding the principles of equality at the global level is articulated as a collective responsibility of the global community [3].

There has been a substantial reduction in child mortality during the Millennium Development Goals era; however, inequalities in progress have been masked by the considerable achievements in terms of the mean magnitude of mortality reduction, both globally and at the country level [4]. Reflecting this, the World Health Organization (WHO) developed a guideline to assess health inequality, thereby disaggregating progress by strata such as socioeconomic status, gender, geographic location, and the like [5].

Health disparities between the least and most deprived groups within a country are relatively well documented [6–18]. Inverse associations between child mortality and socioeconomic status have frequently been reported for disparities within a country [6, 11, 13, 19]. However, trends among socioeconomic strata of a country do not sufficiently indicate health inequalities across the globe. The progress over time and distribution of all cause or cause-specific child mortality between countries by income has not been rigorously monitored, and hence remains unknown.

Eliminating health inequalities encompasses between-country level, and is not restricted to within-country level [19, 20]. The WHO and many scholars have emphasized the importance of tackling health inequality, recognizing it as a matter of justice [5, 21–23]. If we perceive health as a human right, health inequality across the globe should be addressed with the same level of urgency and prioritization as health inequality within a single country.

Simultaneously, investigating the current status of health inequality across countries is critical for informing actors in the global health community so that they can adequately select target countries for effective resource allocation. The global health community has highlighted the need for adequate targeting when selecting partner countries and investigated whether countries were selected in an appropriate manner, particularly for maternal and child health improvements [24–26]. Revealing absolute and relative inequalities in child mortality across countries by income level may highlight the importance of adequately selecting target countries for improving child health.

An equally important implication of exploring absolute and relative inequalities in child mortality is that doing so could have policy implications in terms of accountability by enabling an assessment of whether inequalities in child mortality have converged or diverged during the Millennium Development Goals period. Since a substantial amount of resources has been invested to avert child deaths, such an assessment would hold the global community accountable to inform global citizens and taxpayers whether these global efforts have contributed to a high-level convergence of health inequality around the world [27]. Similarly, not assessing the inequalities between countries around the world challenges the global health community such as bilateral and multilateral donors, academia and NGOs, to develop global interventions and policies to reduce and eliminate unequal child health around the world [14].

Monitoring disparities and the time-trend in child mortalities across the world by income level helps to track the progress towards the global goal of eliminating health inequalities, and to identify adequate global health policies and interventions surrounding global child mortality reduction.

Literature examining time-trends of health inequalities are also predominantly focused on within-country inequalities. Many of the country-level inequality studies found evidence suggesting that inequalities in the uder-5 mortality by wealth strata have been shrinking, diverging or have remained unchanged [14, 19, 28–35].

It is uncertain whether improvements in child survival have been substantial in low income countries, and whether the improvement was proportional to economic growth between 2000 and 2015. Hence, for informing global policy decisions, it is crucial to understand whether the global inequality in under-5 child mortality has been converging or diverging.

To address this knowledge gap, we aimed to analyze for the period 2000–2015, the child mortality disparities between countries by income level and its progression; we further explored whether inequality across the globe by income level has converged over time.

## Methods

### Data source

The Global Burden of Disease and the UN Inter-agency Group for Child Mortality Estimation (IGME) annually assess trends in child mortality using distinct methods [36–43]. Both assessments are highly correlated (0.983) and are frequently used for understanding child mortality estimates [36–43]. The data published by Liu et al. was used for the current study. Liu and colleagues updated the number of cause-specific child deaths for neonates and children aged 1–59 months in 2016 [44]. The number of age-specific deaths was multiplied by cause-specific mortality fractions to estimate the cause-specific child deaths. Age-specific child deaths were assessed by using the child mortality provided by the UN IGME [45] and live births estimated by the UN Population Division [46].

In their study for assessing cause-specific child mortality fractions, the vital registration (VR) data, a VR-based multi-cause model (VRMCM), or a verbal autopsy-based multi-cause model (VAMCM) was used, depending on the presence of VR data and the level of child mortality of a country. VR data were used for neonates in 67 countries and for 1- to 59-month-olds in 69 countries. VRMCM was used for neonates in 47 countries and for 1- to 59-month-olds in 44 countries, where they had inadequate VR and a low rate of child mortality (i.e. less than 35 child deaths per 1000 live births). VAMCM was applied to estimate cause-specific child mortality fractions for neonates in 80 countries and for 1–59-month-olds in 81 countries, in which they had inadequate VR and a high rate of child mortality (i.e. 35 child deaths or more per 1000 live births). The estimation methods developed by Liu and colleagues have been described elsewhere in detail [37, 38, 44]. For the current study, we collected data on gross domestic product per capita in 2000–2015, from the Organization for Economic Cooperation and Development (OECD) database.

### Data analysis

Changes in child mortality rate in the period 2000–2015 have been analyzed in relation to the country’s income at the global level. In this study, the gross domestic product per capita was used to explore the trend of overall and cause-specific child mortality across the world by the income level between 2000 and 2015.

To assess the degree of inequality across countries, four types of measures were used: the difference and the ratio of child mortality rate, the concentration index, and the Erreygers index [47–50]. As a way of assessing absolute inequality, we measured the rate difference between the lowest and highest income quintile. For calculating ratio of the relative measure of inequality, child mortality in the lowest income quintile was divided by the highest income quintile. Details on the confidence interval for these measures are described elsewhere [50, 51].

The concentration index was assessed to quantify the level of relative inequality across all countries by wealth. It was calculated as twice the area between the concentration curve, L(p), and the equality line (the 45-degree line running from the bottom-left corner to the top-right) [49].

Concentration curves were determined for visualizing inequality of all cause and cause-specific child mortality between countries by wealth rank [49–53]. The cumulative proportion of under-5 child deaths (on the y-axis) were plotted against the cumulative percentage of live births ranked by wealth (on the x-axis), beginning with the lowest GDP per capita [19]. If all countries had the same child mortality rate, we would have a 45-degree line of the concentration curve. If child mortality rate is higher in the lower income country, the concentration curve lies above the equality line.

For instance, the concentration index is zero if there is no income-related inequality. When the concentration curve lies above the equality line, the index has a negative value, which indicates that child deaths are disproportionately concentrated among the poor. If it has a positive value, the curve lies below the line of equality, indicating that child mortality is higher among the better-off.

The Erreygers index, a normalized version of concentration index, was also calculated since the value of concentration index is affected by an average of all cause or cause-specific child mortality [53]. The Erreygers index remains constant if child mortality increases in all quintiles by the same absolute value. Confidence intervals around Erreygers indices and concentration indices were calculated using the methods proposed by Kakwani et al. [50].

As with the concentration index, a negative sign of the Erreygers concentration index indicates that child mortality is concentrated among the worst-off, while a positive sign indicates the converse pattern.

For assessing the longitudinal trend of inequality of child mortality rate by wealth ranking, we applied the hierarchical mixed effect analysis to examine whether there was a significant change in slope of under-5 child mortality rate by GDP per capita, between 2000 and 2015 (dependent variable: under-5 child mortality rate).

The multilevel model of all-cause or cause-specific child mortality contains gross domestic product for individual countries as a lower level that is nested within years, which form a higher level. The dependent variable is all-cause or cause-specific child mortality at the country level.

The level 1 model is:

$$ {\boldsymbol{Y}}_{ij}={\boldsymbol{\beta}}_{oj}+{\boldsymbol{\beta}}_{1j}{\boldsymbol{X}}_{ij}+{\boldsymbol{e}}_{ij} $$

where ***Y***_*ij*_ is child mortality for an individual country i (level 1) at year j (level 2); ***X***_*ij*_, gross domestic product per capita of a country i at year j; ***β***_*oj*_, the intercept of the child mortality in year j; ***β***_1*j*_, the slope for the relationship in year j between gross domestic product per capita and child mortality; ***e***_*ij*_, the random errors of prediction for the level 1.

The level 2 model is:

$$ {\boldsymbol{\beta}}_{oj}={\boldsymbol{\gamma}}_{oo}+{\boldsymbol{\gamma}}_{01}{\boldsymbol{W}}_j+{\boldsymbol{u}}_{oj} $$

$$ {\boldsymbol{\beta}}_{1j}={\boldsymbol{\gamma}}_{1o}+{\boldsymbol{u}}_{1j} $$

where the dependent variables are the intercepts and the slopes for the gross domestic product per capita at level 1 (country) in the groups of level 2 (year), and ***γ***_*oo*_ is the overall intercept. This is the grand mean of the child mortality scores across all the groups when all predictors are equal to 0; ***W***_*j*_, the year; ***γ***_01_, the overall regression coefficient, or the slope, between the dependent variable and the year; ***u***_*oj*_, the random error component for the deviation of the intercept of a year from the overall intercept; ***γ***_1*o*_, the overall regression coefficient, or the slope, between the child mortality and the gross domestic product per capita; and ***u***_1*j*_, the error component for the slope, meaning the deviation of the year-specific slopes from the overall slope. We compared the two regression models using the likelihood ratio test (lr test): the first model was set assuming that the slope was the same across the years, and the second model included the assumption of slope change. For the second model, the year was set as a cluster variable in the mixed effects model, and random coefficients of the slope (dependent variable: under-5 child mortality rate; independent variable: GDP per capita) were examined. Chi square of the lr test and *p*-value were used to examine significant slope changes.

We additionally tested whether the slope differed between the years 2000 and 2015 by fitting two random-effects models. The dependent variable was the under-5 child mortality rate and the independent variable was GDP per capita. We allowed the slopes to vary by year in one model and assumed that the slope would be the same across the years in the other model. We examined the results of a likelihood ratio test between the two models.

## Results

**Fig. 1:**
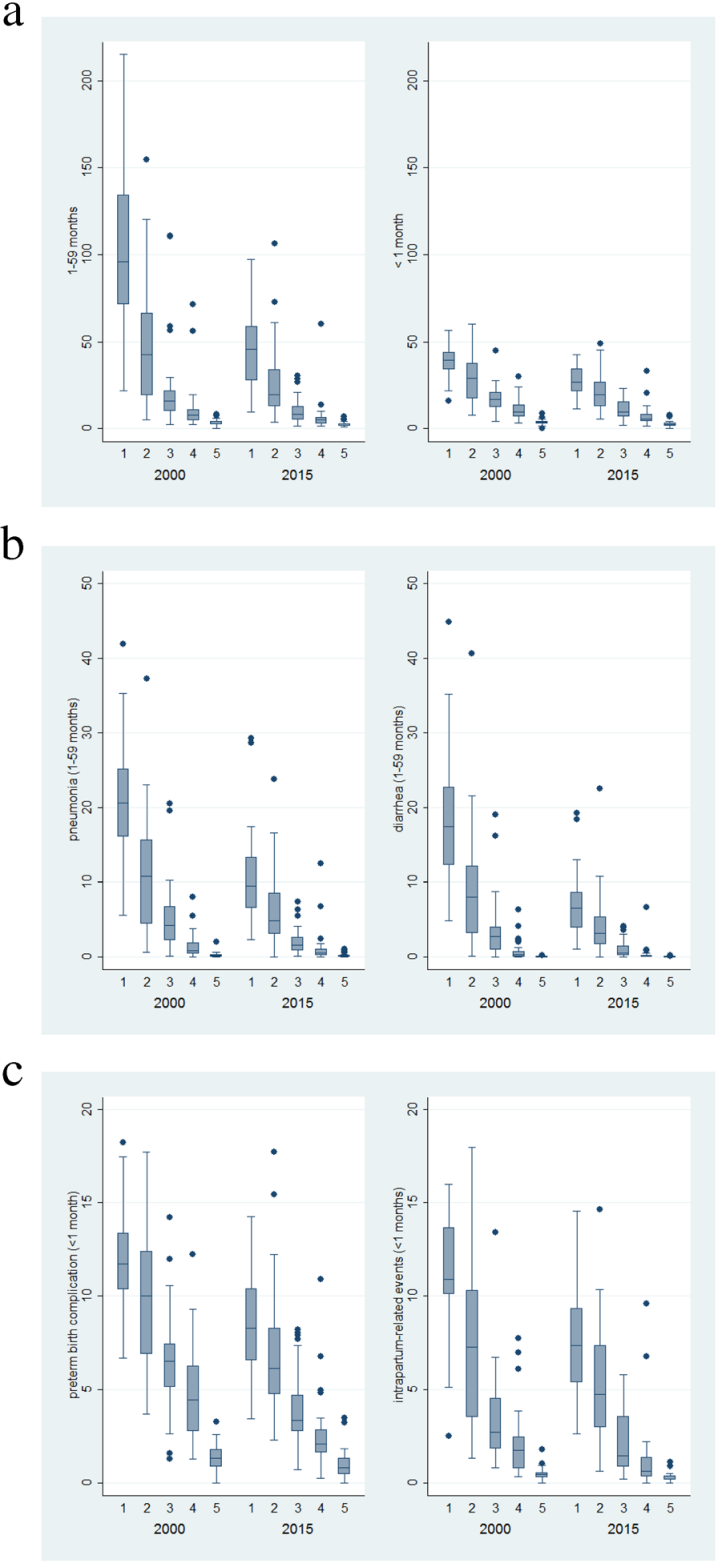
Box plots of child mortality rate in 2000 and 2015 by income quintile (**a**) box plots of post-neonatal (left) and neonatal (right) child mortality rate in 2000 and 2015 by income quintile (x-axis: income quintile; from the lowest to the highest) (**b**) box plots of pneumonia-specific (left) and diarrhea-specific post-neonatal child mortality rate in 2000 and 2015 by income quintile (x-axis: income quintile; from the lowest to the highest) (**c**) box plots of preterm birth complication-specific (left) and intrapartum-related events-specific neonatal child mortality rate in 2000 and 2015 by income quintile (x-axis: income quintile; from the lowest to the highest)

**Fig. 2:**
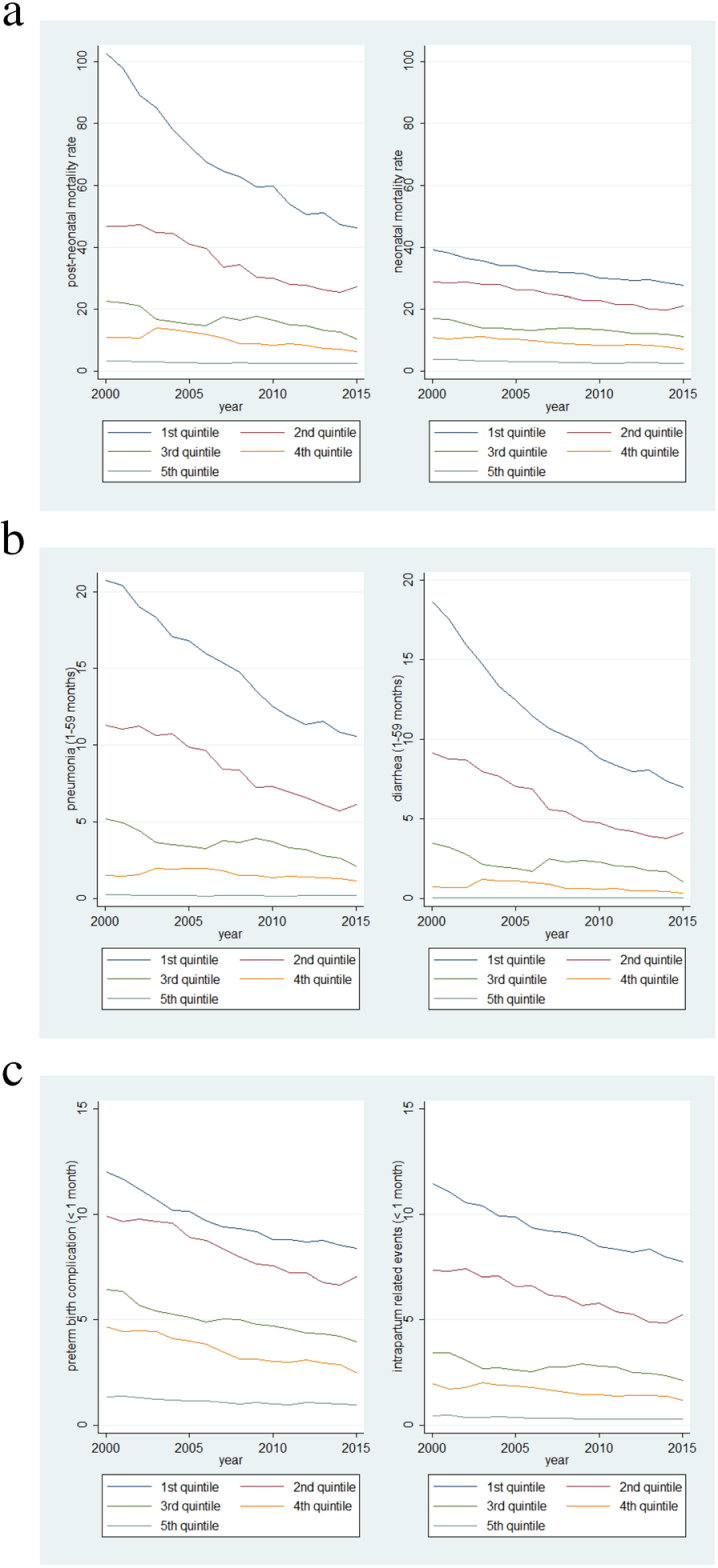
Time trend of child mortality rate from 2000 to 2015 by income quintile (**a**) time trend of post-neonatal (left) and neonatal child mortality rate from 2000 to 2015 by income quintile (**b**) time trend of pneumonia-specific (left) and diarrhea-specific post-neonatal child mortality rate from 2000 to 2015 by income quintile (**c**) time trend of pre-term birth complication specific- (left) and intrapartum-related events specific- (right) neonatal child mortality rate from 2000 to 2015 by income quintile

**Fig. 3:**
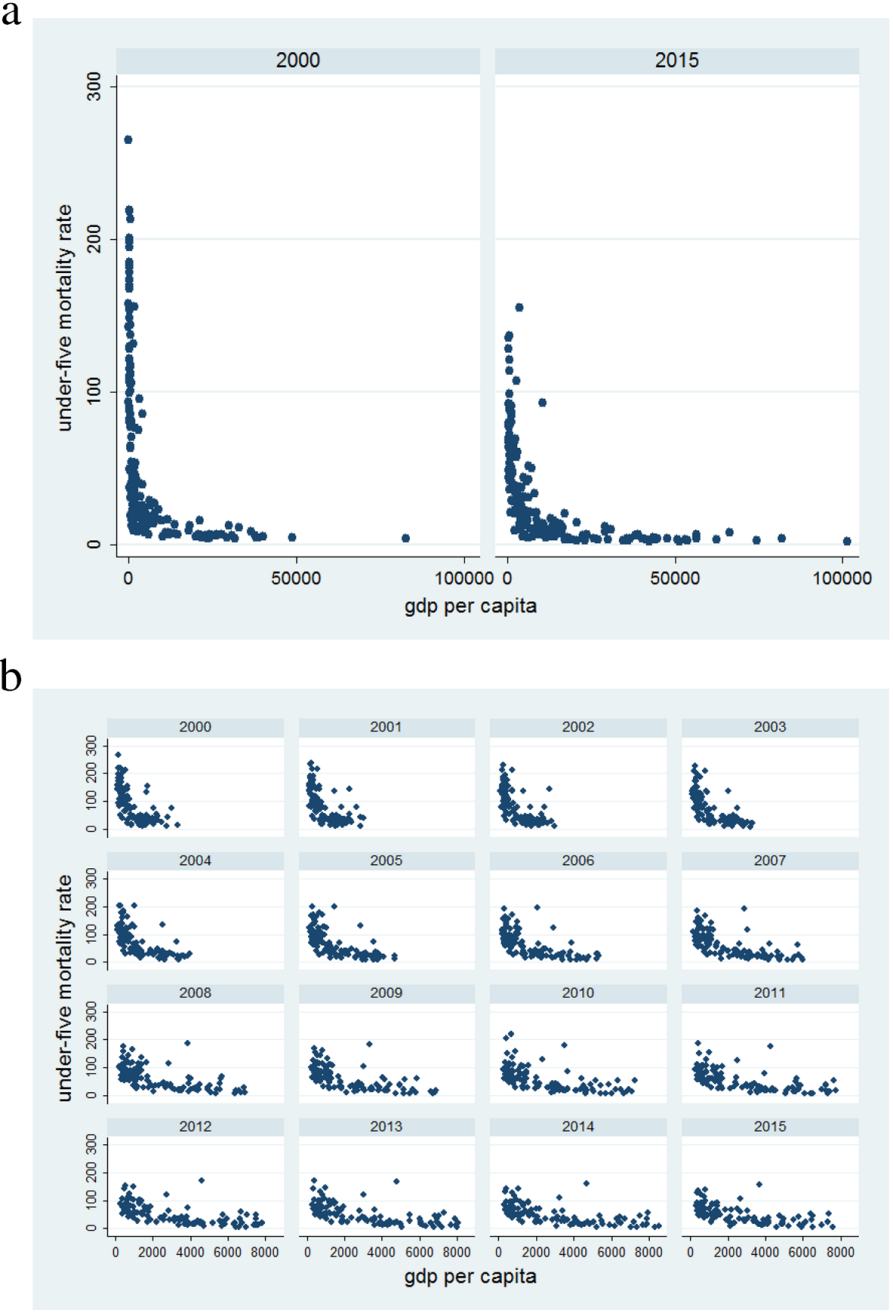
Under-five mortality rate by GDP per capita (**a**) under-five mortality rate by GDP per capita in 2000 and 2015 (among all quintiles) (**b**) under-five mortality rate by GDP per capita in 2000 and 2015 (among 1st, 2nd and 3rd quintiles)

Figure 1 shows a median of child mortality rates between 2000 and 2015, by the wealth quintiles across 194 countries. With no exception, all cause and cause-specific child mortality show a gradient by wealth quintile, where the poorest quintile always had the highest mortality rates, while richest quintile had substantially lower rates. For instance, in the year 2000, mean child mortality rate was 100.6 deaths (Standard Deviation: 43.5) per 1000 live births in the lowest quintile, and 5.6 deaths (SD: 2.8) in the highest quintile. This gradient is still evident in 2015. Figure 2 shows the distribution of under-5 mortality estimates from 194 countries, along with a smoothed (median spline) fit curve for each wealth stratum from 2000 to 2015. The disparity by income level was also observed in all cause-specific child mortality, but the gradient varied across causes (Fig. 2, Additional file 1: Figure S1). Overall, the mortality rate showed gradual decline in the 194 countries. The gradients of child mortality by income quintile were seen in all causes, with faster declines being observed in the most deprived countries. Tables 1 and 2 show inequalities in all cause and cause-specific child mortality rates by applying the four measures of inequality (Table 1 for the rate difference between the richest and poorest countries, and the rate ratio; Table 2 for the concentration index, and the Erreygers index). All the four measures show significant inequalities across countries with respect to income levels (95% confidence interval) (Fig. 3).

**Table 1:** Relative and absolute inequality of child mortality between the first and fifth quintile across the world

	2000					
	1st quintile	5th quintile				
(live births)	(27393198)	(10376885)				
	deaths	deaths	Rate Ratio	95% CI	Rate Difference	95% CI
under-5 child	3,823,853	70,279	20.7	20.5–20.8	132.8	132.7–132.9
neonatal (< 1)	1,108,367	37,414	11.2	11.1–11.4	36.9	36.8–40.0
pneumonia (< 1)	84,934	304	106.0	94.8–118.7	3.0	2.9–3.1
preterm (< 1)	320,923	15,571	7.8	7.7–7.9	10.2	10.1–10.3
intrapartum (< 1)	330,073	3845	32.6	31.6–33.6	11.7	11.6–11.8
sepsis (< 1)	176,629	1906	35.2	33.6–36.8	6.2	6.2–6.4
tetanus (< 1)	51,801	1	19,661.7	2769.6–139,582.4	1.9	1.8–2.0
others (< 1)	65,850	4602	5.4	5.3–5.6	1.9	1.8–2.0
congenital (< 1)	66,550	11,182	2.3	2.2–2.4	1.4	1.3–1.5
diarrhea (< 1)	11,613	0				
post-neonatal (1–59)	2,715,486	32,865	31.4	31.0–31.7	95.9	95.8–96.0
pneumonia (1–59)	558,339	2012	105.3	100.8–110.0	20.2	20.1–20.3
preterm (1–59)	46,371	2242	7.9	7.5–8.2	1.5	1.4–1.6
intrapartum (1–59)	25,564	535	18.1	16.6–19.8	0.9	0.8–1.0
meningitis (1–59)	134,133	998	51.0	47.9–54.3	4.8	4.7–4.9
others (1–59)	290,435	10,429	10.6	10.4–10.8	9.6	9.5–9.7
congenital (1–59)	60,801	9053	2.5	2.4–2.6	1.3	1.3–1.5
diarrhea (1–59)	499,873	877	216.3	202.5–231.2	18.2	18.1–18.3
measles (1–59)	289,853	32	3438.0	2431.3–4861.7	10.6	10.5–10.7
injury (1–59)	121,673	6566	7.0	6.9–7.2	3.8	3.7–3.9
malaria (1–59)	521,587	0				
AIDS (1–59)	139,972	62	856.9	668.0–1099.2	5.1	5.0–5.2
pertussis (1–59)	26,880	51	200.1	152.0–263.3	1.0	0.9–1.1
	1st quintile	5th quintile				
(live births)	(23974652)	(11065174)				
	deaths	deaths	Rate Ratio	95% CI	Rate Difference	95% CI
under-5 child	1,577,598	59,687	12.2	12.1–12.3	60.4	60.2–60.5
neonatal (< 1)	621,951	32,302	8.9	8.8–9.0	23.0	22.9–23.1
pneumonia (< 1)	42,664	222	88.9	44.9–101.4	1.8	1.7–1.8
preterm (< 1)	180,824	13,402	6.2	6.1–6.4	6.3	6.2–6.4
intrapartum (< 1)	172,700	3373	23.7	22.9–24.5	6.9	6.8–7.0
sepsis (< 1)	115,480	1595	33.5	31.9–35.2	4.6	4.6–4.6
tetanus (< 1)	9102	0				
others (< 1)	38,174	4795	3.7	3.6–3.8	1.2	1.1–1.2
congenital (< 1)	59,021	8916	3.1	3.0–3.2	1.7	1.6–1.8
diarrhea (< 1)	3986	0				
post-neonatal (1–59)	955,647	27,385	16.1	15.9–16.3	37.3	37.2–37.4
pneumonia (1–59)	222,300	1735	59.3	56.5–62.1	9.1	9.0–9.2
preterm (1–59)	29,556	1805	7.6	7.2–7.9	1.1	1.0–1.2
intrapartum (1–59)	16,180	446	16.8	15.3–18.4	0.6	0.5–0.7
meningitis (1–59)	39,757	435	42.3	38.5–46.5	1.6	1.5–1.7
others (1–59)	185,750	9494	9.0	8.9–9.2	6.9	6.8–7.0
congenital (1–59)	41,546	7198	2.7	2.6–2.8	1.1	1.0–1.2
diarrhea (1–59)	142,927	649	101.9	94.3–110.0	5.9	5.8–6.0
measles (1–59)	20,102	1	9297.2	1309.6–66,004.5	0.8	0.7–0.9
injury (1–59)	97,074	5470	8.2	8.0–8.4	3.6	3.5–3.7
malaria (1–59)	112,194	1				
AIDS (1–59)	30,148	6	2323.9	1044.0–5173.1	1.3	1.2–1.4
pertussis (1–59)	18,106	142	59.0	50.0–70.0	0.7	0.6–0.8

**Table 2:** Concentration index

	2000		2000		2015		2015	
	Concentration index	95% CI	Erreyger’s index	95% CI	Concentration index	95% CI	Erreyger’s index	95% CI
All cause	−0.33	− 0.32,-0.34	− 0.41	− 0.39,-0.43	− 0.3	− 0.29,-0.32	− 0.37	− 0.35,-0.39
neonatal	−0.25	− 0.24,-0.26	− 0.48	− 0.46,-0.51	− 0.28	− 0.27,-0.30	− 0.43	− 0.41,-0.46
pneumonia	−0.24	− 0.23,-0.25	− 0.56	− 0.53,-0.58	− 0.33	− 0.32,-0.35	− 0.43	− 0.41,-0.45
preterm	−0.21	− 0.20,-0.22	− 0.48	− 0.45,-0.51	− 0.26	− 0.25,-0.28	−0.35	− 0.33,− 0.37
intrapartum	-0.3	− 0.29,-0.31	− 0.5	− 0.48,-0.52	− 0.32	− 0.31,-0.34	− 0.43	− 0.41,-0.45
sepsis	−0.33	− 0.32,-0.35	− 0.49	− 0.46,-0.51	− 0.37	− 0.35,-0.39	−0.48	− 0.45,-0.50
tetanus	−0.39	− 0.37,-0.41	− 0.21	− 0.20,-0.22	− 0.38	− 0.36,-0.41	− 0.14	− 0.13,-0.15
others	−0.14	− 0.13,-0.15	− 0.39	− 0.37,-0.41	− 0.19	− 0.18,-0.20	− 0.34	− 0.33,-0.36
congenital	−0.06	− 0.05,-0.06	− 0.21	− 0.20,-0.23	−0.15	− 0.14,-0.16	− 0.29	− 0.27,-0.31
diarrhea	−0.36	− 0.34,− 0.38	-0.38	− 0.36,-0.40	− 0.35	− 0.33,-0.37	− 0.25	− 0.24,-0.27
post	−0.39	− 0.37,-0.41	− 0.36	− 0.34,-0.38	− 0.32	−0.30,− 0.34	-0.34	−0.32,− 0.36
pneumonia	-0.36	−0.35,-0.38	− 0.4	− 0.38,-0.43	− 0.34	− 0.32,-0.36	− 0.29	− 0.27,-0.31
preterm	− 0.32	− 0.31,-0.34	− 0.37	− 0.35,-0.39	− 0.29	− 0.28,-0.31	− 0.26	− 0.25,-0.28
intrapartum	− 0.36	− 0.34,-0.38	− 0.42	− 0.39,-0.44	− 0.33	− 0.31,-0.35	− 0.31	− 0.29,-0.32
meningitis	−0.46	− 0.44,-0.48	− 0.26	− 0.24,-0.27	− 0.39	− 0.37,-0.41	− 0.21	− 0.20,-0.22
others	−0.27	− 0.25,-0.28	− 0.42	− 0.39,-0.44	− 0.29	−0.28,-0.31	− 0.27	− 0.26,-0.29
congenital	−0.03	− 0.02,-0.03	− 0.21	− 0.20,-0.24	− 0.08	− 0.07,-0.08	− 0.18	− 0.17,-0.20
diarrhea	− 0.42	− 0.40,-0.43	− 0.34	− 0.32,− 0.36	-0.36	− 0.34,-0.38	− 0.26	− 0.24,-0.27
measles	− 0.58	− 0.55,-0.61	− 0.17	− 0.16,-0.18	− 0.38	−0.36,-0.40	− 0.06	−0.06,-0.06
injury	−0.18	− 0.17,-0.19	− 0.47	−0.45,-0.49	− 0.27	− 0.26,-0.29	− 0.42	−0.40,-0.44
malaria	−0.67	−0.63,-0.70	− 0.21	−0.19,-0.23	− 0.42	−0.39,-0.45	− 0.18	−0.17,-0.20
AIDS	−0.43	−0.40,-0.46	− 0.09	−0.08,-0.10	− 0.37	−0.34,-0.40	− 0.14	−0.13,-0.15
pertussis	−0.4	−0.38,-0.42	− 0.58	−0.55,-0.61	− 0.38	−0.36,-0.41	− 0.53	−0.50,-0.56

Compared to the children in the least deprived socioeconomic quintile, the mortality rate for children in the most deprived socioeconomic quintile was nearly 20.7 times higher (95% Confidence Interval: 20.5–20.8) in 2000 and 12.2 times (95% CI: 12.1–12.3) higher in 2015. The overall post neonatal child mortality rate in the lowest income quintile was 31.4 times (95% CI: 31.0–31.7) higher in 2000 compared with that in the highest income quintile, and the relative disparity across income quintile converged to 16.1 times (95% CI: 15.9–16.3) higher in 2015.

The relative and absolute inequality of child mortality between the first and fifth quintile across the world have declined over time for all diseases, but is observed to be more pronounced in infectious diseases (pneumonia, diarrhea, measles, and meningitis). The rate ratio of pneumonia- and diarrhea-specific childhood mortality associated with wealth has decreased substantially. In 2000, post-neonatal children in the first quintile had 105.3 times (95% CI: 100.8–110.0) and 216.3 times (95% CI: 202.5–231.2) higher risks of pneumonia- and diarrhea-specific child mortality, respectively, than children in the fifth quintile. In 2015, the corresponding rate ratios were 59.3 times (95% CI: 56.5–62.1) and 101.9 times (95% CI: 94.3–110.0) higher. However, compared to non-communicable disease, infectious diseases still show far more severe disparity with respect to income quintiles.

Table 3 shows results of the likelihood ratio test of the two models between slope changes and no slope changes of child mortality rate by GDP per capita for the period 2000–2015. There were significant slope changes in almost all cause-specific child mortalities except for preterm birth complication and intrapartum-related events, suggesting significant reduction occurred in the lowest income countries. Mixed effects analysis demonstrates the convergence of under-5 mortality in the 194 countries across income level.

**Table 3:** Change in inequality of child mortality between countries by income

*model*: *dependent: child mortality (log); independent: gdp* per capita	log-linear regression (by year)								*generalized linear mixed effect (multilevel longitudinal model; cluster: year)*	
	2000				2015				likelihood test of random slope	
	coefficient	95% CI	*p*-value	adjusted R square	coefficient	95% CI	*p*-value	adjusted R square	chi(2)	*p*-value
under-5 child	−28.2	(−31.6, − 24.8)	< 0.001	0.60	− 17.2	(− 19.4, − 15.0)	< 0.001	0.58	30.21	< 0.001
neonatal (< 1)	−7.8	(−8.5, −7.0)	< 0.001	0.69	−6.3	(−7.1, −5.6)	< 0.001	0.63	27.52	< 0.001
pneumonia (< 1)	−0.6	(− 0.7, − 0.5)	< 0.001	0.69	− 0.5	(− 0.5, − 0.4)	< 0.001	0.60	30.33	< 0.001
preterm (< 1)	− 2.4	(− 2.6, − 2.1)	< 0.001	0.67	− 1.9	(− 2.1, − 1.6)	< 0.001	0.57	58.87	< 0.001
intrapartum (< 1)	−2.4	(− 2.6, − 2.1)	< 0.001	0.64	−1.9	(− 2.1, − 1.6)	< 0.001	0.58	12.29	< 0.001
sepsis (< 1)	− 1.3	(− 1.5, − 1.2)	< 0.001	0.61	− 1.2	(− 1.4, − 1.1)	< 0.001	0.61	9.97	0.002
tetanus (< 1)	− 0.3	(− 0.4, − 0.2)	< 0.001	0.28	−0.1	(− 0.2, − 0.1)	< 0.001	0.18	12.11	< 0.001
others (< 1)	− 0.4	(− 0.5, − 0.3)	< 0.001	0.56	− 0.3	(− 0.4, − 0.2)	< 0.001	0.48	26.52	< 0.001
congenital (< 1)	− 0.3	(− 0.3, − 0.2)	< 0.001	0.28	− 0.4	(− 0.5, − 0.3)	< 0.001	0.43	16.53	< 0.001
diarrhea (< 1)	− 0.1	(− 0.1,− 0.1)	< 0.001	0.44	-0.1	(− 0.1, − 0.1)	< 0.001	0.35	11.10	< 0.001
post-neonatal (1–59)	− 20.4	(− 23.3, − 17.6)	< 0.001	0.53	−10.8	(− 12.4, − 9.25)	< 0.001	0.50	24.85	< 0.001
pneumonia (1–59)	− 4.4	(− 5.0, − 3.9)	< 0.001	0.61	− 2.6	(− 3.0,-2.2)	< 0.001	0.49	24.13	< 0.001
preterm (1–59)	− 0.3	(− 0.4, − 0.3)	< 0.001	0.44	− 0.3	(− 0.3, − 0.2)	< 0.001	0.45	0.47	0.49
intrapartum (1–59)	− 0.2	(− 0.2, − 0.1)	< 0.001	0.48	− 0.2	(− 0.2, − 0.1)	< 0.001	0.50	0.57	0.45
meningitis (1–59)	− 1.0	(1.1, − 0.9)	< 0.001	0.47	− 0.4	(− 0.5, − 0.4)	< 0.001	0.39	22.41	< 0.001
others (1–59)	− 2.2	(− 2.5, − 2.0)	< 0.001	0.59	−2.1	(− 2.4, − 1.8)	< 0.001	0.50	4.67	0.03
congenital (1–59)	− 0.3	(− 0.4, − 0.2)	< 0.001	0.31	− 0.4	(− 0.4, − 0.3)	< 0.001	0.31	7.15	0.01
diarrhea (1–59)	− 4.0	(− 4.5, − 3.4)	< 0.001	0.52	−1.7	(−2.0, − 1.5)	< 0.001	0.44	31.59	< 0.001
measles (1–59)	−1.8	(−2.3, − 1.3)	< 0.001	0.06	− 0.2	(− 0.3, − 0.1)	< 0.001	0.22	12.66	< 0.001
pertussis (1–59)	− 0.2	(− 0.23, − 0.1)	< 0.001	0.48	− 0.2	(− 0.2, − 0.1)	< 0.001	0.53	3.10	0.08

**Fig. 4:**
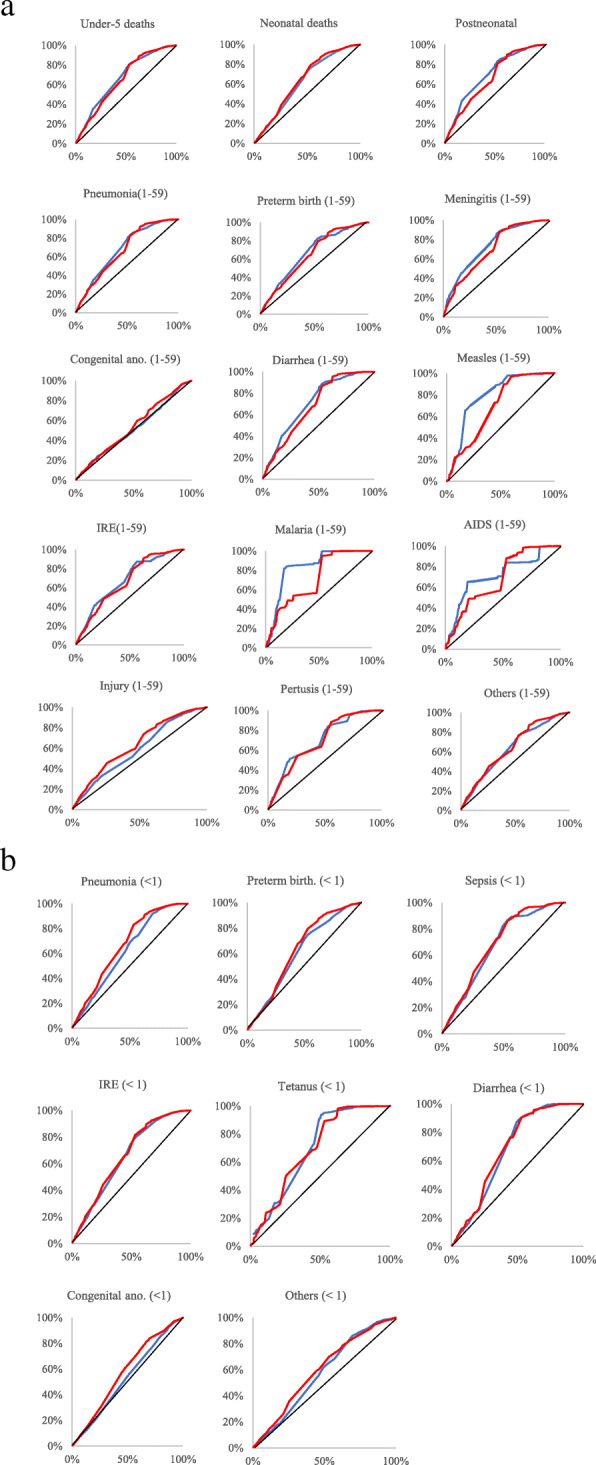
Concentration curve (**a**) all cause under-five/neonatal/post-neonatal deaths and cause-specific post-neonatal deaths (**b**) cause-specific neonatal deaths (blue line: concentration curve in 2000, red line: concentration curve in 2015, black diagonal line: equality line; y-axis: accumulated percentage of deaths, x-axis: accumulated percentage of live births from the lowest wealth ranking to the highest; < 1: neonates, 1-59: 1-59 months (post-neonates); Congenital ano.: congenital anomaly, preterm birth.: preterm birth complication, IRE: intrapartum-related events)

Figure 4 illustrates relative inequalities across 194 countries with the concentration curves. The concentration curves are always above the equality line, suggesting that child mortality is concentrated in the lower income countries for post neonatal mortality, the concentration curves in 2015 appear to be closer to the line of equality compared to those in 2000, suggesting the mortality gap has reduced. However, the concentration curves for neonatal mortality have increased distance from the equality line over the same time period, demonstrating that the inequality of neonatal mortality has worsened over the years, particularly in the 2nd, 3rd, and 4th wealth quintile.

## Discussion

In this study, we used four measures to assess inequalities in child mortality rates between 194 countries by income levels. Child mortality rates in the poorest group are always higher than the richest group for all cause and cause-specific child mortalities. Overall, the risk difference and rate ratio in child mortality rate between the lowest and highest income group has shrunk with respect to cause-specific child mortalities. The inequality in child mortality between countries by income declined in 2000–2015, but a huge disparity still remains.

Results for the temporal fashion of child mortality in this study demonstrate a grand convergence across the income quintile around the world. The linear mixed effect analysis model suggests that time trends of child mortality reduction are significantly different across income quintiles. Though there were decreasing trends in all cause and cause-specific child mortalities during the period, the rate declined faster in the most deprived quintile than in the other group, which contributed to closing the gap between countries by income level.

The gradients across income quintile were more pronounced in infectious diseases in 2000. The cause-specific child mortalities declined more sharply in the lowest income group particularly in pneumonia, diarrhea, and measles, which might probably be due to substantial efforts and investments made by the global health community [27]. Considerable investments have been made during this 15-year period, largely in combating infectious diseases. More than 100 global health initiatives (GHIs) were created after the Millennium Development Goals were declared, such as the President’s Emergency Plan for AIDS Relief (PEPFAR) [27]. GHIs have been sometimes criticized because their vertical approach tended not to strengthen, or even to weaken, countries’ health systems [27]. However, this study has demonstrated that GHIs should be duly appreciated because the pronounced convergence in child mortality caused by infectious diseases can, in part, be explained by these intensive efforts. This study also implies that we should rigorously re-assess the pros and cons of GHIs with respect to their contributions towards strengthening health systems.

The findings of our study may not be surprising, since the countries with the highest child mortality burdens—a group in which low-income countries predominate—might be more likely to reduce their mortality rate than countries with the lowest burdens. However, we cannot take for granted the accelerated progress in the lowest-income countries for the following reasons. Without a strengthened health system, it has been reported that substantial reductions in child mortality can hardly be achieved [27, 54]. Comprehensive interventions built on a strengthened health system are crucial for preventing deaths from pneumonia or diarrhea, as such interventions include behavioral changes, inter-sectoral collaboration, and environmental improvements [55–59]. For this reason, we infer that the substantial reduction in the low-income countries might have been attributable to strengthened health systems, although this possibility still needs to be rigorously investigated. If this suggestion is true, we would expect that progress will be maintained or accelerated during the Sustainable Development Goals period.

An equally important point is that we urge the global community to pay full attention to the fact that there are tremendous inequalities between better-off and worst-off countries in child mortality due to infectious diseases, even though mortality caused by these conditions is relatively easy to avoid with existing interventions. The rates of pneumonia-specific neonatal mortality and diarrhea-specific post-neonatal mortality among children living in the lowest-income countries are still close to or above 100 times of that among those in the highest--income countries. The situation is even worse for tetanus, measles, and AIDS. Child deaths due to these causes are almost or totally zero in the highest-income countries, while they are still causing numerous deaths of children in the lowest-income countries. All in all, these facts clearly show that there is still much room for improvement in child health in infectious diseases. A silver lining is that we may be able to accelerate progress in child mortality reduction because these diseases can be tackled with existing interventions. This study sheds light on the global movement surrounding the Countdown to 2030 Initiative to increase essential health coverage, particularly in the 81 countries with highest burden of maternal and child deaths. The integrated Global Action Plan for Pneumonia and Diarrhea (GAPPD) initiative of the World Health Organization to avoid pneumonia- and diarrhea-specific child deaths needs to receive careful, ongoing attention around the globe [60].

Jamison and colleagues [54] presented the prospect of a bright future with a grand convergence of health across the world. This study reveals that child health has converged continuously in the past 15 years with the help of global efforts, although there is still a long way to go.

The collective efforts made by the global health community need to be duly commended across the globe, and this optimistic story needs to be disseminated and maintained during the Sustainable Development Goals period.

At the same time, it is important to understand that full convergence was not found for all-cause and cause-specific neonatal mortality rates by wealth ranking, and that the magnitude of the convergence varied across causes. A noteworthy aspect of this study is that it suggests that inequality was more profound among neonates than among post-neonatal children in both 2000 and 2015 based on the Erreygers index, which gives the global health community another reason to place additional focus on this group.

Numerous studies have been conducted on health inequality within individual countries by socioeconomic status, and the concentration of ill health or mortality among the poor has been well documented. However, the evidence for unequal distributions of child mortality by income group between countries remains scarce, and few studies have assessed inequalities in cause-specific child mortality even through within-country comparisons. Clearly, this study urges the global health community to invest more in the lowest-income countries to speed up the grand conversion of child health.

You and colleagues estimated the prospects for child mortality reduction in 195 countries by 2030 based on a worst-case scenario, a best-case scenario, and the SDG target scenario [36]. This study implies that the best-case or SDG target scenario may not be realized without resolving the disproportionately heavy concentration of all-cause and cause-specific child deaths among neonates in the lowest-income countries. Bhutta and colleagues [61] have argued that official development assistance investment can be more effectively utilized when targeting the worst-off groups based on a cost-effect analysis. This study urges global actors to adequately select target countries for global child health programs.

The main limitation of this study is the large gap in data source for the countries. We used cause-specific child mortality published by Liu et al. [44]. Uncertainties and misclassifications are inherent in their data, particularly for the estimates based on a verbal autopsy. Only a small proportion of the global cases of child deaths are medically certified, which hinders precise estimation of cause-specific child mortality. To ensure greater validity of the estimates, Liu and colleagues increased the data points in their updated reports in 2016 (i.e., 2004 data points for vital registration for neonates and 1364 for 1–59-month-olds; 124 for verbal autopsy for neonates and 218 for 1–59-month-olds). A range of organizations, including the World Health Organization, have applied these estimates, although the accuracy of verbal autopsy and vital registration remains to be improved. The estimates model should continue to be refined to create reliable cause-specific child mortality estimates. Investigating the factors behind the between-country inequalities is beyond the scope of this study, and therefore future studies are warranted to bridge the knowledge gap.

## Conclusion

To the best of our knowledge, this study is the first to globally assess inequalities in cause-specific child mortality and its time-trend considering income levels. Reducing health inequalities is the core goal of the global health community. Though there are numerous reports showing persistent disparity of child mortality rates within countries, they do not reveal the inequalities between countries and its temporal fashion. Therefore, the findings in this study have important policy making implications at the global level, by unmasking health inequalities by wealth.

The relative disparity in child mortality across wealth quintiles reduced from 2000 to 2015. The gradients by income quintile were pronounced mostly in infectious disease in 2000, but decreased substantially in 2015. This study helps to close the knowledge gap by assessing progress in health inequalities in terms of all cause and cause-specific child mortalities between countries and their temporal changes. We were able to explore the magnitude of inequalities in cause-specific child mortalities between countries with respect to income levels, and how inequalities change over time. This study helps us to understand where and which diseases should be prioritized by the global community.

Grand convergence in child mortality, particularly in post neonatal children, suggests that the global community has witnessed success to some extent in controlling infectious diseases. However, there has been no meaningful progress in terms of equality across the globe in neonatal child mortality, and enormous disparities still exist for communicable and non-communicable diseases. Efforts to close the gaps must be the priority of the global health community during the period of the Sustainable Development Goals.

## Supplementary information

**Additional file1: Figure S1. **(a) time trend of meningitis-specific child mortality rate from 2000 to 2015 by income quintile (d) time trend of injury-specific child mortality rate from 2000 to 2015 by income quintile (f) time trend of congenital anomaly-specific child mortality rate from 2000 to 2015 by income quintile (g) time trend of pertussis-specific child mortality rate from 2000 to 2015 by income quintile

## Data Availability

The datasets generated and/or analysed during the current study are available here: https://www.thelancet.com/cms/10.1016/S0140-6736(16)31593-8/attachment/5d910574-af99-4748-b5bb-f750a45cd1a7/mmc1.pdf

## Abbreviations

GBD: Global Burden of Disease
GDP: Gross Domestic Product
IGME: Inter-Agency Group for Child Mortality Estimation
OECD: Organization for Economic Cooperation and Development
VR: vital registration
VRMCM: VR-based multi-cause model
