# Supplementary material for: Have inequalities in all-cause and cause-specific child mortality between countries declined across the world?
Source: Int J Equity Health. 2019 Dec 31;19:1. doi: 10.1186/s12939-019-1102-3 (PMC6938619; doi:10.1186/s12939-019-1102-3)
Supplement: Supplementary file 1 — Additional file1: Figure S1. (a) time trend of meningitis-specific child mortality rate from 2000 to 2015 by income quintile (d) time trend of injury-specific child mortality rate from 2000 to 2015 by income quintile (f) time trend of congenital anomaly-specific child mortality rate from 2000 to 2015 by income quintile (g) time trend of pertussis-specific child mortality rate from 2000 to 2015 by income quintile [file 12939_2019_1102_MOESM1_ESM.docx]

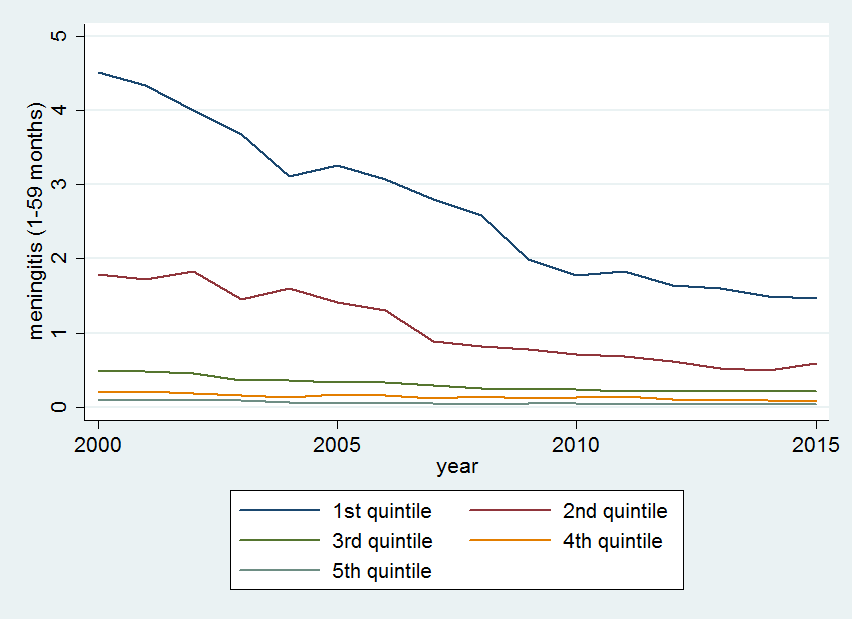


Figure S1 (a) time trend of meningitis-specific child mortality rate from 2000 to 2015 by income quintile


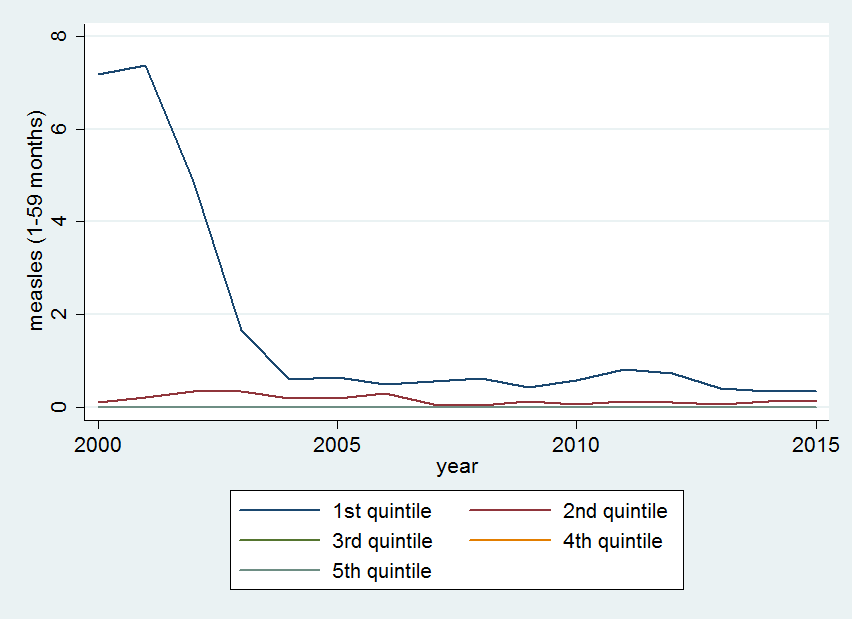


Figure S1 (b) time trend of measles-specific child mortality rate from 2000 to 2015 by income quintile


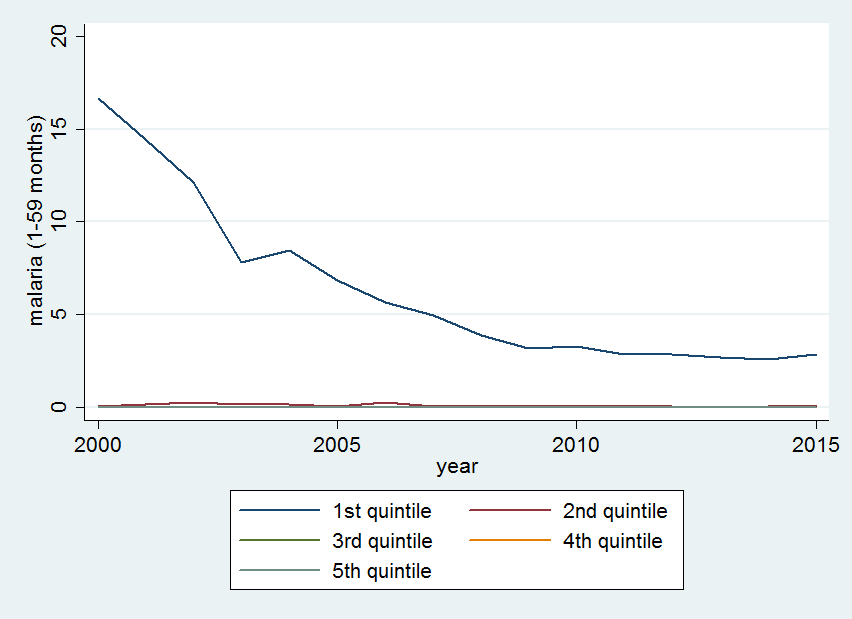


Figure S1 (c) time trend of malaria-specific child mortality rate from 2000 to 2015 by income quintile


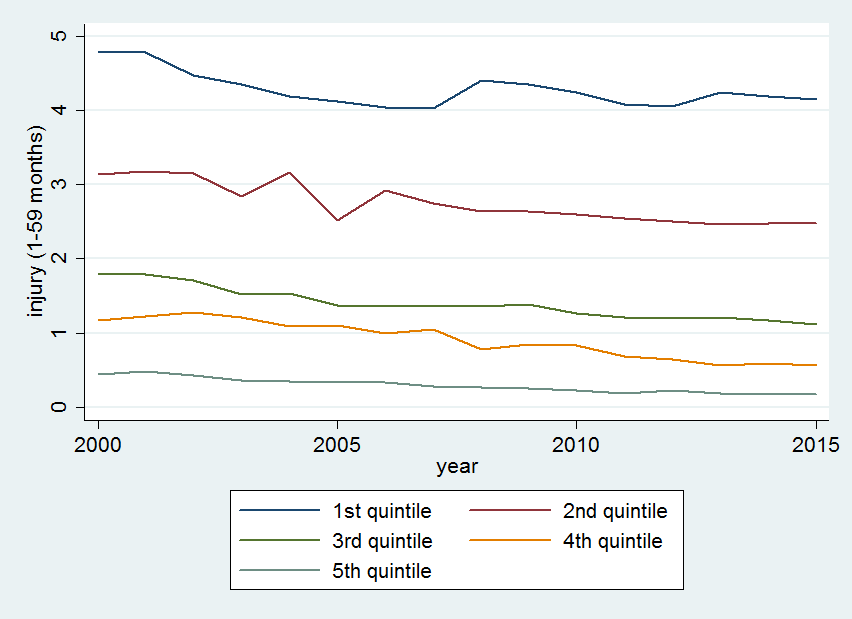


Figure S1 (d) time trend of injury-specific child mortality rate from 2000 to 2015 by income quintile


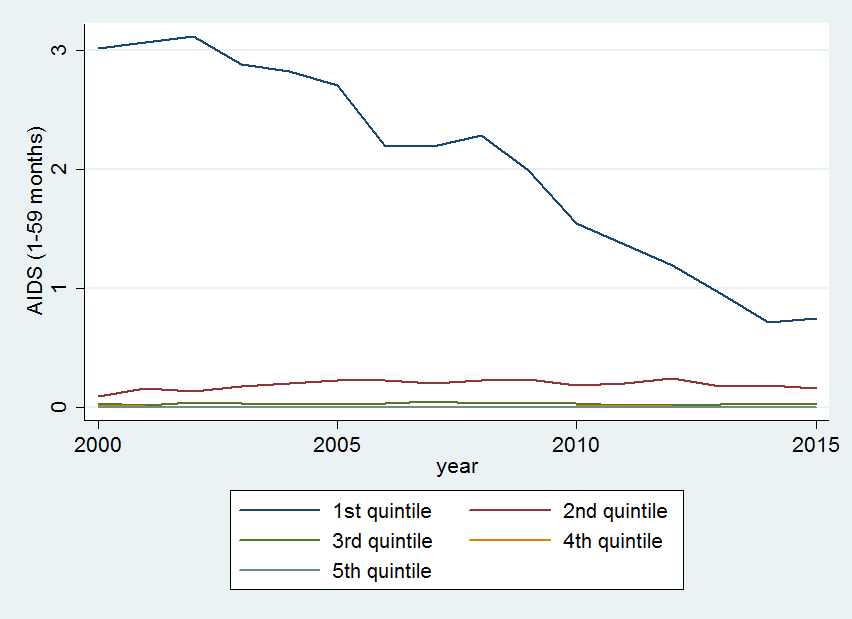


Figure S1 (e) time trend of AIDS-specific child mortality rate from 2000 to 2015 by income quintile


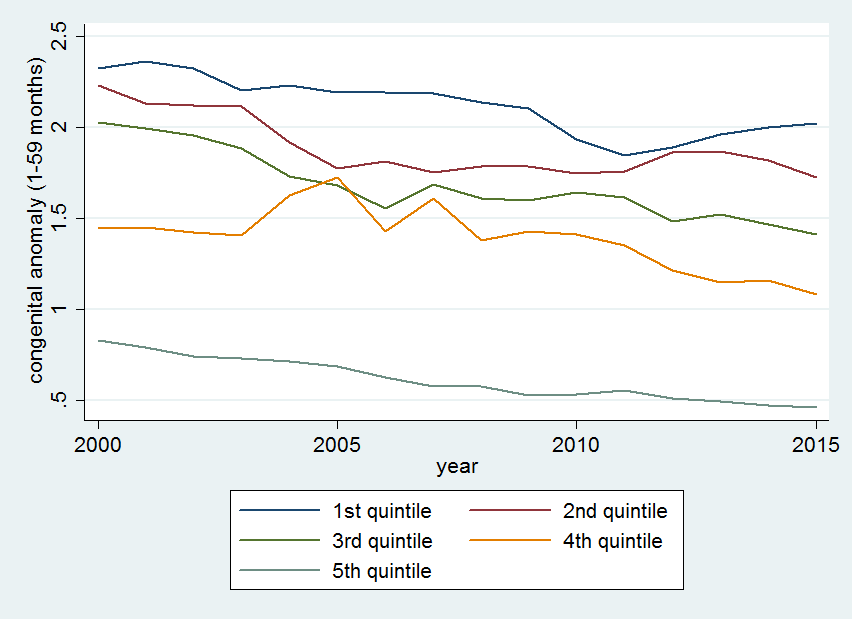


Figure S1 (f) time trend of congenital anomaly-specific child mortality rate from 2000 to 2015 by income quintile


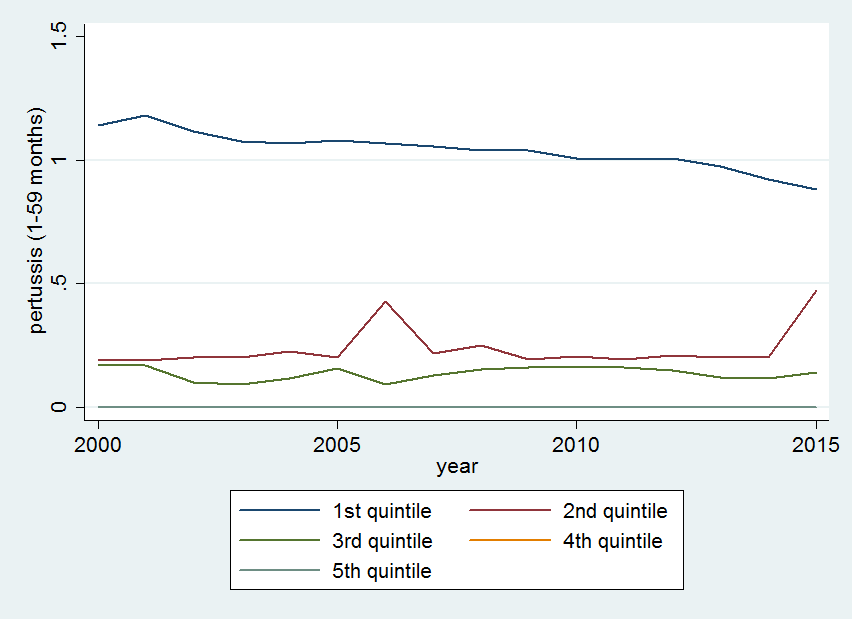


Figure S1 (g) time trend of pertussis-specific child mortality rate from 2000 to 2015 by income quintile
